# Perspective Adjunctive Therapies for COVID-19: Beyond Antiviral Therapy

**DOI:** 10.7150/ijms.51935

**Published:** 2021-01-01

**Authors:** Ping Ho, Jing-Quan Zheng, Chia-Chao Wu, Yi-Chou Hou, Wen-Chih Liu, Chien-Lin Lu, Cai-Mei Zheng, Kuo-Cheng Lu, You-Chen Chao

**Affiliations:** 1 Division of General Surgery, Department of Surgery, Taipei Tzu Chi Hospital, Buddhist Tzu Chi Medical Foundation, New Taipei City 231, Taiwan.; 2 Division of Pulmonary Medicine, Department of Internal Medicine, School of Medicine, College of Medicine, Taipei Medical University, Taipei 11031, Taiwan.; 3 Graduate Institute of Clinical Medicine, College of Medicine, Taipei Medical University, Taipei, Taiwan.; 4 Division of Pulmonary Medicine, Department of Internal Medicine, Shuang Ho Hospital, Taipei Medical University, New Taipei City 23561, Taiwan.; 5 Division of Nephrology, Department of Medicine, Tri-Service General Hospital, National Defense Medical Center, Taipei 114, Taiwan.; 6 Division of Nephrology, Department of Medicine, Cardinal-Tien Hospital, School of Medicine, Fu-Jen Catholic University, New Taipei City 234, Taiwan.; 7 Division of Nephrology, Department of Medicine, Taipei Hospital, Ministry of Health and Welfare, New Taipei City 242, Taiwan.; 8 Division of Nephrology, Department of Medicine, Fu Jen Catholic University Hospital, School of Medicine, Fu Jen Catholic University, New Taipei City 242, Taiwan.; 9 Division of Nephrology, Department of Internal Medicine, Taipei Medical University Shuang Ho Hospital, 235 New Taipei City, Taiwan.; 10 Division of Nephrology, Department of Internal Medicine, School of Medicine, College of Medicine, Taipei Medical University, 110 Taipei, Taiwan.; 11 Taipei Medical University-Research Center of Urology and Kidney (TMU-RCUK), Taipei Medical University, 110 Taipei, Taiwan.; 12 Division of Nephrology, Department of Medicine, Taipei Tzu Chi Hospital, Buddhist Tzu Chi Medical Foundation, New Taipei City 231, Taiwan.; 13 Division of Gastroenterology, Department of Internal Medicine, Taipei Tzu Chi Hospital, Buddhist Tzu Chi Medical Foundation, New Taipei City 231, Taiwan.; 14 School of Medicine, Tzu Chi University, Hualien 970, Taiwan.

**Keywords:** COVID-19, traditional medicines, statins, melatonin, indomethacin, vitamins and minerals

## Abstract

The coronavirus disease 2019 (COVID-19) pandemic is the largest health crisis ever faced worldwide. It has resulted in great health and economic costs because no effective treatment is currently available. Since infected persons vary in presentation from healthy asymptomatic mild symptoms to those who need intensive care support and eventually succumb to the disease, this illness is considered to depend primarily on individual immunity. Demographic distribution and disease severity in several regions of the world vary; therefore, it is believed that natural inherent immunity provided through dietary sources and traditional medicines could play an important role in infection prevention and disease progression. People can boost their immunity to prevent them from infection after COVID-19 exposure and can reduce their inflammatory reactions to protect their organ deterioration in case suffering from the disease. Some drugs with in-situ immunomodulatory and anti-inflammatory activity are also identified as adjunctive therapy in the COVID-19 era. This review discusses the importance of COVID-19 interactions with immune cells and inflammatory cells; and further emphasizes the possible pathways related with traditional herbs, medications and nutritional products. We believe that such pathophysiological pathway approach treatment is rational and important for future development of new therapeutic agents for prevention or cure of COVID-19 infection.

## Introduction

The coronavirus disease 2019 (COVID-19) outbreak is the greatest major threat experienced by the human race in living memory 1, 2. As of October 2020, over 44 million reported cases of COVID-19 and over 1 million deaths around the world have been noted and are still rapidly increasing. As this COVID-19 pandemic continues, the understanding and active surveillance of high-risk population, along with taking appropriate preventive measures, has become critical. Compared to previous coronaviruses, severe acute respiratory syndrome (SARS)-coronavirus (CoV)-1 and Middle East respiratory syndrome (MERS)-CoV, SARS-CoV-2, which is responsible for COVID-19, has higher transmissibility, even in those with milder or no symptoms 3. The number of secondary cases per infected individual varies among different regions and is estimated to range from 2.0 to 2.5 4. This has been suggested to be related to different viral epidemiologic characteristics, viral viability and infectivity in aerosols and surroundings 5.

Patients with SARS-CoV-2 experience a clinical spectrum, from mild/asymptomatic forms to acute respiratory distress syndrome (ARDS) requiring ventilator support and admission to an intensive care unit (ICU). Clinical symptoms vary and include respiratory involvement to damage to multiple organs, including the heart, liver, and kidney and organs of the gastrointestinal tract, among others. Ten to twenty percent of symptomatic patients experience disease progression and 3-5% require intensive care unit (ICU) admission 6. Elderly patients more than 60 years of age and those who are male, obese and have underlying comorbid conditions (e.g., hypertension, diabetes mellitus, chronic respiratory disorders, etc.), tend to have poor clinical outcomes 7. Unfortunately, SARS-CoV-2 infection is still out of control, and no effective drug or vaccine is currently available. In this review, we discuss the pathophysiology of COVID-19, possible adjunctive therapies for COVID-19 and reasonable therapies for different comorbid conditions beyond the antiviral medications.

## Novel pathophysiological pathways associated with COVID-19

**Figure 1:**
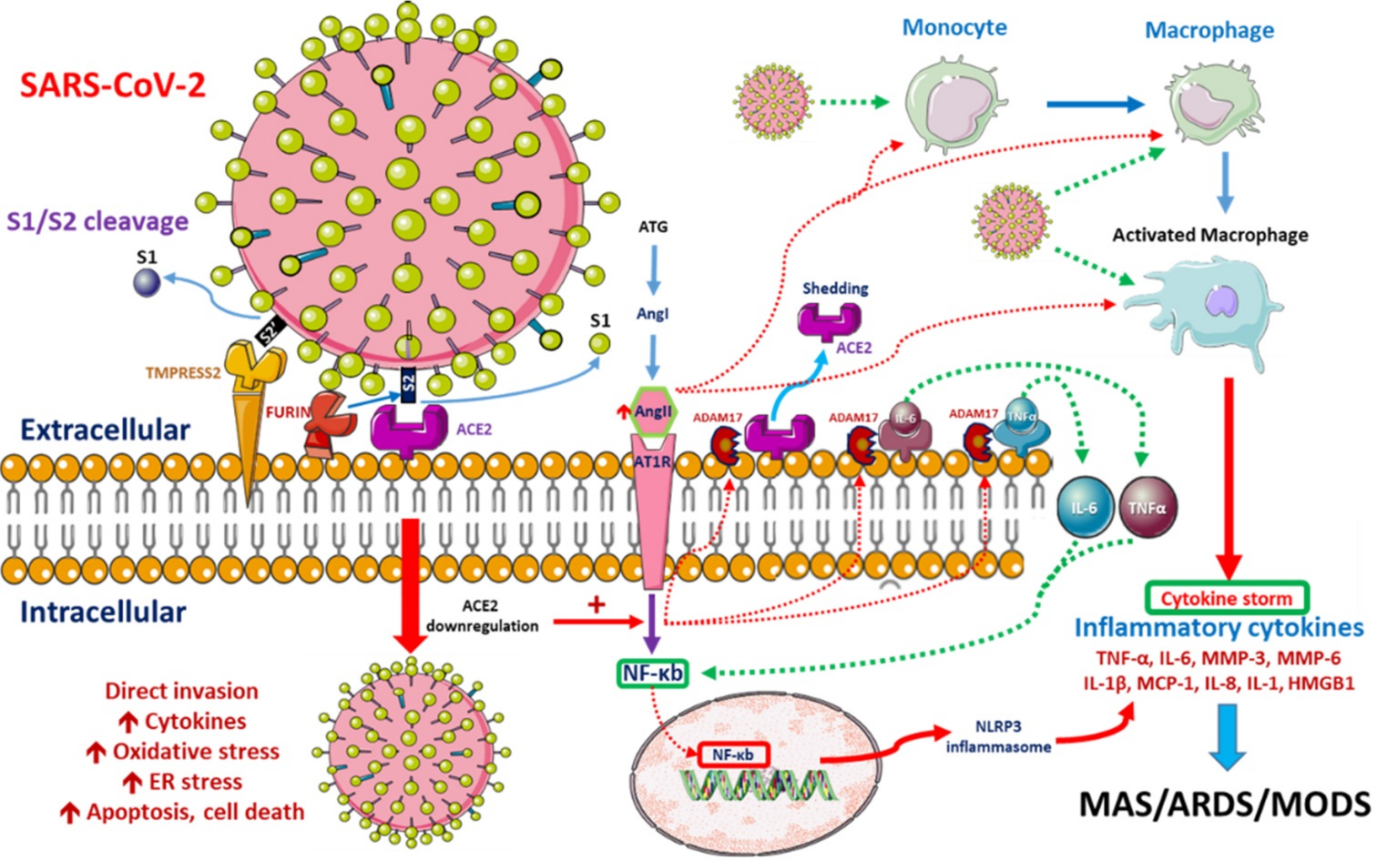
** Possible pathophysiological pathways after SARS-CoV-2 entry.** When SARS-CoV-2 enters cells, its surface spike (S) glycoprotein must be cleaved at two different sites by host cell proteases. ACE2-dependent entry at the cell membrane is triggered by S protein cleavage performed by host proteases furin and/or TMPRSS2. Intracellular activation of S protein is mediated by cathepsins in lysosomes and/or by furin in the trans-Golgi network (TGN) 8, 9. SARS-CoV-2 replication is inhibited by the synthetic furin inhibitor 10. After entry into the host cell, the virus downregulates ACE2 expression, which in turn upregulates Ang II. Upregulated Ang II interacts with its receptor, AT1R, and modulates the gene expression of several inflammatory cytokines via NF-κB signaling. This Ang II/AT1R interaction also promotes macrophage activation, which produces the inflammatory cytokines that may cause ARDS or MAS. Some metalloproteases, such as ADAM17, shed these proinflammatory cytokines and ACE2 receptors to the soluble form, which aids in the loss of the protective function of surface ACE2 and may aggravate SARS-CoV-2 pathogenesis 11. SARS-CoV-2-infected monocytes and macrophages produce large amounts of numerous types of proinflammatory cytokines and chemokines, which contribute to local tissue inflammation and a dangerous systemic inflammatory response known as a cytokine storm. Both local tissue inflammation and the cytokine storm play fundamental roles in the development of COVID-19-related complications, such as acute respiratory distress syndrome (ARDS), which is a main cause of death in COVID-19 patients 12. *Abbreviations:* AngII: angiotensin II, ARDS: adult respiratory distress syndrome, ATG: angiotensinogen, MAS: macrophage activation syndrome, MODS: multiple organ dysfunction syndrome.

SARS-CoV-2 enters the human lung, heart and kidney cells through the binding of its spike (S) proteins to angiotensin-converting enzyme-2 (ACE2) receptors on host cells 13, 14. It has been demonstrated that SARS-CoV-2 has 10- to 20-fold higher affinity for ACE2 than SARS-CoV-1, which might be related to its higher contagiousness 15. ACE2 downregulation after the binding of SARS-CoV-2 to ACE2 impairs downstream angiotensin-II (Ang-II) metabolism. The resultant Ang-II accumulation induces pulmonary vasoconstriction, acute lung injury, lung edema and eventual lung fibrosis 16-18. Severe COVID-19 cases are characterized by endothelial dysfunction and hypercoagulability states, resulting in thrombosis and pulmonary embolism 19. Progressive pulmonary microvasculature damage further enhances viral invasion and destruction 20. Possible pathological pathways after SARS-CoV-2 entry are depicted in **Figure 1.**

Patients with severe CoV-19 infection further experience a systemic inflammatory response and acute respiratory distress syndrome (ARDS), which results from “cytokine storm syndrome” 21, an aberrant interaction among interferons, interleukins, chemokines, etc. As a result, severe COVID-19 patients express higher amounts of proinflammatory cytokines, including interleukin (IL)-6, IL-10, IL-1β, monocyte chemoattractant protein 1 (MCP1) and tumor necrosis factor (TNF)-α 22, 23. In addition, SARS-CoV-2-infected lung epithelial cells produce IL-6 and IL-8, potent chemo-attractors for both neutrophils and T cells 24, resulting in further destruction.

A principal defense against SARS-CoV-2 involves both cell and antibody mediated immunity. Although serum antibodies are detected for approximately 2 weeks in all patients 25, their longevity and long-term protection is still unknown. T-cell responses against the SARS-CoV-2 spike protein correlate well with serum antibody titers in COVID-19 patients and are being clinically applied for the development of novel vaccines 26, 27. The infiltration of these cells into the lungs and other organs further destroys and exaggerates COVID-19 severity 28, 29. Innate immunity is conferred primarily by epithelial cells, alveolar macrophages and dendritic cells (DCs), which fight against the virus as first line immunity 24 until adaptive immunity is activated. The infiltration of neutrophils potentiates innate immunity 30, 31 and induces lung injury. T regulatory lymphocyte (Treg) levels were demonstrated to be significantly reduced in severe COVID-19 patients 32. As infection progresses, the activation and tissue infiltration of T cells with concomitant reduction in circulating T cells (CD4+ T cells, CD8+ T cells) occurs, which determines the severity of COVID-19 33. Interestingly, aberrant pathological cytotoxic T cells derived from CD4^+^ T cells are noted among severe COVID-19 patients 34. The infiltration of these cytotoxic T cells and inflammatory monocytes exaggerates lung destruction 35. The expression of IFN-γ by CD4+ T cells also tends to be lower in severe cases than in moderate cases 32. Apart from known anti-inflammatory pathways, drugs and traditional medicines with immunomodulatory, antiviral, antithrombotic, anti-cytokine and antifibrotic properties are being studied for possible use in COVID-19 patients.

Heme oxygenase (HO-1) is an antioxidant enzyme that cleaves heme into carbon monoxide, ferrous iron and biliverdin, and these downstream products limit inflammation and oxidative stress 36, 37. Patients with comorbid conditions related to metabolic syndrome (diabetes, obesity, hypertension, cardiovascular disease) generally have lower HO-1 levels and increased susceptibility to inflammation 38-41 and are more prone to the triggering of cytokine storms related to COVID-19 42. This might explain the increased susceptibility and higher mortality and morbidity among these patients. A Taiwanese study revealed an association between higher HO-1 expression in the HO1 (-497A/*) genotype and lower susceptibility to SARS coronavirus infection 43. Clinically, medications and herbs that exert anti-inflammatory and cytoprotective effects by increasing HO-1 include resveratrol (RESV; tri-hydroxyl stilbene), pterostilbene 44-46, curcumin 47, 48, HMG CoA reductase inhibitors (statins) 49, 50 and melatonin 51, 52.

**Figure 2:**
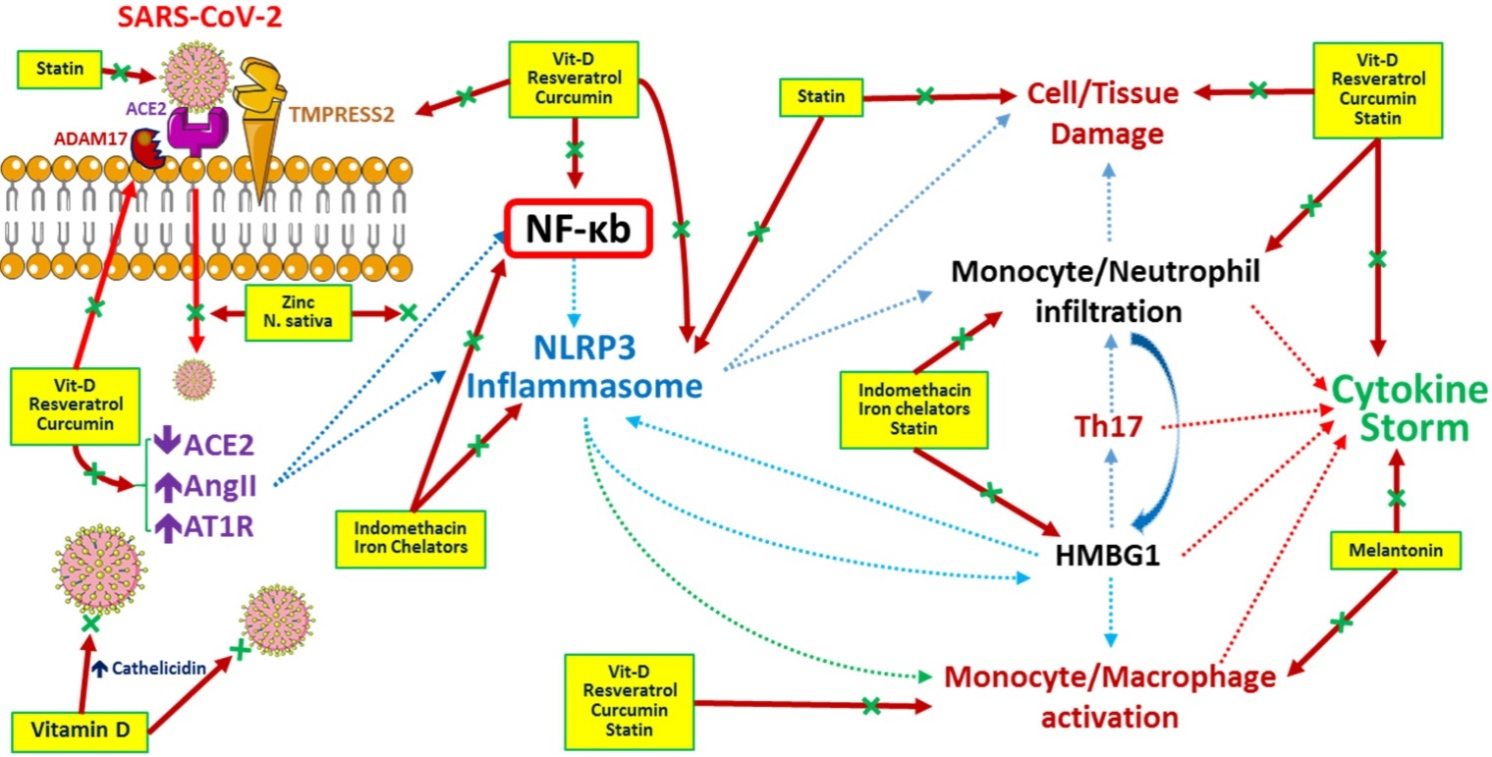
Potential pathways regulated by medications and/or herbs in treatment/prevention of COVID-19 infection. Resveratrol (stilbene-based natural compounds), curcumin and statins inhibit the viral entry and destruction, improve the cell/tissue damage by preventing inflammatory cells infiltration and reduce cytokine storm related with COVID-19 infection. N. sativa (black seed) and Zinc (Zn) inhibit viral entry and NF-kb related inflammatory pathways. Melatonin inhibits monocyte/ macrophage activation and inflammatory cytokines release. Indomethacin and iron chelators responsible for blocking NF-kb and NLRP3 related inflammatory pathways and inhibiting inflammasome formation. Vitamin D inhibits COVID-19 viral entry, activates ACE2 and cathelicidin production, decreases inflammatory pathways, reduces inflammatory mediators release and improves cytokine storm. Ascorbic acid (Vitamin C) acts as a potent antioxidant with immunomodulatory actions especially by activation of immune cells. Zinc enhancing the Zinc (Zn) uptake by cells and enhancing human immunity. *Abbreviations:* HMBG1: High mobility group box 1, NF-κb: nuclear factor-κB, NLRP3: NOD-, LRR- and pyrin domain-containing protein 3.

Generally, therapeutic options for SARS-CoV-2 infection should focus on vaccines to enhance immunity and medications to modulate the immune system, suppress inflammation, disturb virus-cell interactions, inhibit viral replication, etc. At present, no vaccine is available against COVID-19. Infected patients are currently treated with antivirals, anti-inflammatories, herbal medicines, plasma exchange, etc. (**Table 1**). The possible underlying mechanisms regulated by these medicines during the COVID-19 era are depicted in **Figure 2.**

## Possible adjunctive therapies for COVID-19

### Medications and Herbs with Antiviral, Anti-inflammatory and Antioxidant activities

#### Resveratrol and stilbene-based natural compounds

Plant polyphenols, nonflavonoids and flavonoids are found abundantly in grapes, red wine, mulberry and peanuts and are used as health remedies for their antioxidant, antitumor and antiviral properties 53. Resveratrol (RESV) is a stilbene based nonflavonoid polyphenol that modulates inflammation and exerts reno- and neuroprotective effects through its antioxidant and anti-inflammatory properties 54-57. RESV is demonstrated to be neuroprotective in several ways, including stimulating the Nrf2 pathway and reducing NF-kB activity 58, activating Sirt1 signaling in neurons 59, etc. Nrf2, a member of the cap'n'collar (CNC) basic-leucine-zipper transcription factors, and its signaling are also of interest for COVID-19 patients because Nrf2 serves as an anti-inflammatory, anti-apoptotic, and antioxidant factor through different pathways 60.

In addition, RESV exerts antiviral effects in viral infections through several mechanisms, such as the activation of ERK1/2 signaling 61, the promotion of cell proliferation and the enhancement of SIR1 signaling 62, which subsequently improve cellular survival and DNA repair 63. RESV also reduces Middle East respiratory syndrome coronavirus (MERS-CoV) viral-induced apoptosis 64, 65 and decreases inflammation by interfering with the NF-κB pathway 66, 67. A recent study demonstrated the novel use of RESV in the treatment of COVID-19 68, indicating that the effects of RESV may occur through the disruption of the SARS-CoV-2 spike protein and the inhibition of the human ACE2 receptor complex 68. Although RESV in supplemental doses is considered safe, future clinical and experimental studies are still needed to evaluate the dose, efficacy, and safety of resveratrol in fighting against COVID-19.

#### Curcumin

Another natural phenol mostly studied for its antiviral effects is curcumin, a phenolic acid extracted from the rhizome of *Curcuma longa* Linn (family Zingiberaceae). It is a yellow pigment of turmeric and a primary component of curry and the flavanol found in green tea. Curcumin improves neurodegenerative disorders by increasing NF-E2-related factor 2 (Nrf2)/heme oxygenase 1 (HO-1) protein expression and decreasing the apoptosis of PC12 cells 69. Similar to other plant phenols, curcumin possesses anti-inflammatory and immunomodulatory activities, as well as anticancer, anti-arthritic and anti-atherosclerosis effects 47. Previous data have shown that curcumin exerts antiviral activities against the human immunodeficiency virus (HIV), herpes simplex virus, Chikungunya virus, Zika virus, hepatitis and adenovirus 70-72. From molecular docking studies of SARS-CoV2 viral proteins and potential antiviral agents, curcumin inhibits the entry of SARS-CoV2 by binding to the viral S protein and the viral attachment sites of the ACE2 receptor protein 73. Similarly, the antithrombotic, anti-cytokine and antifibrotic properties of curcumin may benefit severe COVID-19 patients 74, 75. Further, intranasal application of curcumin may effectively prevent SARS-CoV2 infection through inhibiting the local ACE2 receptor. Despite robust preclinical data, there is still no known safety dose or availability of cucurmin among COVID-19 patients. Therefore, well-controlled studies are crucial for assessing any possible curcumin benefit in COVID-19.

#### *N. sativa* (black seed) and zinc (Zn)

*N. sativa* L. (black seed) has been used primarily as a traditional medicine for a variety of diseases, including skin diseases, bronchial asthma, gastrointestinal disorders, toothache, diabetes mellitus, hypertension, etc. 76-78, for more than 2000 yr (16). Recently, many studies have demonstrated that *N. sativa* seeds not only have antioxidant 79, anti-inflammatory, immunomodulatory and antitumor properties 80, 81 but also possess antimicrobial 82, antidermatophytic 83 and anti-cytomegalovirus 84 properties. It has been demonstrated that *N. sativa* L. improves lung inflammation by promoting a balance between pro- and anti-inflammatory pathways through modulatory actions on interleukin-4 (IL-4) and interferon-γ 85. *N. sativa* oil exhibits antiviral effects by promoting the upregulation of both innate immunity (splenic M & phi expression) and adaptive immunity (the stimulation of the CD4+ T lymphocytes) in a murine cytomegalovirus model 84. Black seed oil supplementation also decreases viral load and improves biochemical parameters in individuals with hepatitis C virus (HCV) infection 86. From these findings, the possible therapeutic potential of *N. sativa* seed and its active compounds are being extensively studied in COVID-19 patients. A recent molecular-docking-based study 87 revealed that active ingredients of *N. sativa*, nigellidine and α-hederin, serve as natural inhibitors of SARS-CoV-2 87.

Recently, *N. sativa* and Zn combined supplementation in COVID-19 patients was proposed on the basis of the hypothesis that the bioactive agents of* N. sativa*, thymoquinone and nigellimine block SARS-CoV-2 entry into pneumocytes, enhance zinc (Zn) uptake by cells and enhance human immunity. The immune-boosting activities of Zn are pleiotropic and participate in the activation of adaptive and acquired immunities, as well as intracellular antioxidant activities. On the other hand, the availability of Zn to pneumocytes is crucial in COVID-19 patients because Zn inhibits viral entry, blocks polyprotein processing 88, and inhibits recombinant SARS-CoV virus RNA-dependent RNA polymerase (RdRp) activity 89. Thus, combined *N. sativa* and Zn supplementation might represent a complementary approach to antiviral treatment against COVID-19 and needs additional clinical trials.

#### HMG CoA reductase inhibitors (statins)

Since patients at greater risk for SARS-CoV-2 infection have common comorbidities, whether their routine medications are associated with COVID-19 severity is an interesting topic. Among these, statins, which are lipid-lowering drugs, are considered possible agents for their pleiotropic anti-inflammatory effects. The cholesterol within lipid rafts in the viral envelope is important for viral entry of coronaviruses, including SARS-CoV, and depleting cholesterol results in a significant reduction in viral mRNA 78. Lipid rafts play an important role in developing new therapies, and statins disturb viral binding by decreasing cholesterol and disrupting lipid rafts. Moreover, statins exert an anti-inflammatory effect by inhibiting the toll-like receptor (TLR)-MYD88-NF-kB pathway 90. This anti-inflammatory effect is independent of the cholesterol-lowering effect and is also supported by clinical studies 91. However, the efficacy of statins as an immunomodulatory treatment for viral infections is still controversial. Earlier observational studies suggested that statin therapy reduced mortality in influenza viral infections 92. It also demonstrated that statin therapy was related to reduced H1N1 severity in hospitalized patients 93. However, sensitivity analysis concluded that the mortality improvement in response to statins was related to unmeasured confounding factors 94. On the other hand, a multicenter clinical trial revealed that patients with infection-induced ARDS on statin therapy exhibited higher IL-18 levels, related deterioration due to ARDS and increased mortality 95.

Another consideration for statin therapy in COVID-19 patients is its antithrombotic activity. Markedly elevated D-dimer levels and a high risk of thrombogenicity were noted among critically ill COVID-19 patients 96-98. In the JUPITER trial, statins have been demonstrated to significantly decrease the rate of deep vein thrombosis 99. Statins exert antithrombotic actions through various mechanisms, such as reduced tissue factor expression, decreased platelet aggregation, and increased endothelial cell thrombomodulin expression 100. However, reliable evidence for statin use in COVID-19 is still lacking, and more clinical trials, focusing on patient population, drug effectiveness, type, dosage, etc., are required.

#### Melatonin

Melatonin, the primary pineal gland hormone, is a regulator of circadian and seasonal rhythms that is known for its anti-inflammatory and immunomodulatory actions 101, 102. Melatonin influences neutrophils, CD4 T cells, CD8 T cells, and B cells through membrane melatonin receptors 103 and modulates the production and release of various cytokines 101. Geng-Chin et al. 104 revealed that melatonin protected against ventilator-related lung injury through the upregulation of IL-10 production. Similar studies on protective effects in the lung were also related to melatonin related circadian immunity and anti-inflammatory effects 105, 106.

Based on recent data, COVID-19 most severely affects those aged 60 years' and above, males, and those with comorbid conditions 7. Since lower plasma melatonin concentration is noted in individuals at approximately age 60, in males and in different comorbid conditions 7, 107, 108, the susceptibility of these populations to severe COVID-19 might be related to their increased melatonin requirement. Melatonin is generally safe for both short- and long-term use at a pharmacological oral dose of 2 mg once a day in the early evening. A recently proposed prophylactic protocol for melatonin use in COVID-19 patients is 2 mg oral melatonin once a day immediately after contact with an infected person. Combined use with other long-term medications, such as antihypertensive agents, oral hypoglycemic agents and anti-inflammatory drugs, appears safe 109.

#### Indomethacin

Indomethacin is a potent anti-inflammatory agent that non-selectively inhibits cyclooxygenase (COX)-1 and -2 enzymes and is mostly used to treat inflammatory conditions. Indomethacin exerts anti-inflammatory actions through the inhibition of TNF, IL-6 and superoxide free radicals 110. Since indomethacin inhibits COX-2 and viral protein synthesis 111, antiviral activities against different viruses have been explored, including herpes virus 6 112, cytomegalovirus 113, hepatitis B virus 114, etc. Thus, recent *in vivo* and *in vitro* studies consider the use of indomethacin in COVID-19 era. Amici et al revealed that in *in vitro* studies of monkey VERO cells (SARS-CoV) and in *in vivo* studies of dogs (canine coronavirus, CCoV), indomethacin significantly reduced both SARS-CoV and CCoV by inhibiting viral RNA synthesis 115. In contrast, other authors revealed potential severe and late complications related to the use of ibuprofen in COVID-19 patients 116, 117. Thus, novel use of indomethacin in this COVID-19 era should be balanced with its gastrointestinal, renal, and hematological side effects. Given the cost and availability of indomethacin, RCT trials on outpatients or on patients with documented SARS-CoV-2 infection without cytokine storm should be further studied.

#### Iron chelators

Excess intracellular iron may promote excessive oxidative and nitrosative stress, which may correlate with ARDS and pulmonary fibrosis 118, 119. Generally, hyperferritinemia is common during systemic inflammation. A biological protein sequence study found that SARS-CoV-2 protein sequence forms a complex with porphyrin within hemoglobin and dissociates the heme iron, resulting in increased free iron levels among severe COVID-19 patients 120. Increased serum ferritin levels may represent a marker of disease progression or a key modulator of a vicious cycle of events that contributes to a hyperinflammatory condition and further tissue damage 121. Iron chelators, including deferoxamine (DFO), not only reduce iron overload but also possess immunomodulatory and anti-inflammatory actions 122. In addition, DFO decreases IL-6 and endothelial inflammation 123, the most important mechanism related to multiorgan damage and failure among COVID-19 patients 123. Therefore, iron-chelating agents are believed to improve clinical outcomes and systemic manifestations of COVID-19. However, large RCTs are needed to evaluate the dosage, efficacy, and safety of iron-chelating agents as adjunctive therapy in COVID-19.

## Vitamins and minerals associated with immunomodulatory activities

### Vitamin D

Vitamin D deficiency is a known pandemic and global public health problem that varies based on age, ethnicity, and latitude. Aged, obese, and dark-skinned populations have higher risk for vitamin D deficiency. Environmental factors, including a lack of or reduced r sun (UV-B) exposure, living with air pollution and smoking, are attributed to vitamin D deficiency. The presence of comorbid conditions, such as sepsis, diabetes mellitus, chronic respiratory disease, and cancer, are closely related to vitamin D deficiency 124. Interestingly, in this COVID-19 pandemic, a similarity in prevalent areas and the nature of SARS-CoV-2 infection and vitamin D deficiency was observed 125, which might explain the importance of vitamin D supplementation in COVID-19 7.

An analysis of community outbreaks of COVID-19 has revealed that regions with certain latitude, temperature, and humidity are more prone to the spread of infection. The virus is thermolabile, and less sunlight, lower temperatures and decreased humidity seem to be favorable for COVID-19 126, 127. Clinical studies found a close relationship between vitamin D deficiency and vulnerability to COVID-19 in various populations. A negative association was noted between mean vitamin D levels and COVID-19 mortality in European countries. Regional variation was also noted with the parallel increase in vitamin D deficiency and COVID-19 severity. Inhabitants of southern European countries have lower vitamin D levels than inhabitants of northern countries, where a higher number of COVID-19 cases and higher fatality have been noted. In the United States, a disproportionately higher mortality from COVID-19 has been noted among African Americans (AAs) 128, 129, and the top 3 comorbid conditions among patients who died of COVID-19 are hypertension, diabetes, and chronic kidney disease. It has been demonstrated that a higher prevalence of vitamin D insufficiency is noted among AAs than other Americans 130, 131 due to reduced vitamin D production and insufficient supplementation. This population also tends to have a higher incidence of comorbid conditions, including cardiovascular disease, diabetes, osteoporosis and certain cancers, than whites 130. This calls attention to the relationship between vitamin D deficiency and the severity of COVID-19 and the importance of vitamin D supplementation as possible adjuvant therapy.

Our previous study demonstrated that vitamin D supplementation efficiently increased serum antimicrobial cathelicidin levels, which paralleled increased serum 25-hydroxyvitamin D (25D) levels in uremic hyperparathyroidism patients 132. In other words, vitamin D supplementation increased circulating 25D levels, which improved innate immunity by entering circulating monocytes, increasing local 1,25 dihydroxy-vitamin D (1,25D) production and inducing the production of the antimicrobial peptides cathelicidin and β-defensin 133. Cathelicidin inhibits viral entry and elicits an antiviral status in infected host cells in inhibiting viral replication 134. Defensins directly inhibit viral particles and indirectly inhibit viral entry 135. Mounting evidence suggests that defensins are also responsible for innate immunity, including the activation of T cells, the recruitment and differentiation of macrophages and dendritic cells, and the release of proinflammatory cytokines 136. Vitamin D supplementation also modulates adaptive immunity in individuals with COVID-19 through different pathways. First, vitamin D suppresses the maturation and antigen presentation of dendritic cells. Second, vitamin D increases cytokine production by T- helper cells and promotes the efficiency of T-reg lymphocytes. Last, vitamin D suppresses the cytokine storm and related tissue destruction. This suggests that adequate vitamin D supplementation both attenuates COVID-19-induced immunosuppression and enhances anti-inflammatory actions.

Adequate vitamin D supplementation is also required for reducing RAS activity and increasing ACE2 concentrations in acute lung injury. In other words, adequate vitamin D supplementation induces the ACE2/Ang‐(1‐7) axis and suppresses the renin and ACE/Ang II/AT1R axis 137. Similarly, the increased ACE2 was responsible for viral entrapment and inactivation. Clinically, vitamin D deficiency was found to be associated with an increased incidence of COVID-19 138. Linking the supplementation of vitamin D to improved COVID-19 outcomes is still impossible in this pandemic. Since vitamin D in therapeutic doses is unlikely to harm and may even prevent disease progression in these patients, it should be recommended to all COVID-19 patients. Vitamin D deficiency might be correctable, and supplementation is considered safe and is easily accessible 139. People at risk of COVID-19 should consider supplementation with high doses of vitamin D 10,000 IU daily for a few weeks to rapidly achieve therapeutic levels of 25(OH)D, followed by 5000 IU/day, with the goal of a 25(OH)D concentration of ~40-60 ng/ml 140. Future randomized studies are needed to examine vitamin D doses in COVID-19 patients with different underlying conditions.

### Ascorbic acid (vitamin C)

Vitamin C, especially at higher doses, acts as a potent antioxidant with immunomodulatory actions and significantly accumulates within immune cells 141. Possessing such a powerful antioxidant to clear ROS-related cellular toxicity may indicate the use of vitamin C as adjuvant therapy in symptomatic COVID-19 patients. For example, vitamin C decreases the duration and severity of cold symptoms 142. Several studies are underway to understand the possible mechanisms of action and dose of vitamin C supplementation in individuals with COVID-19. A phase II study is evaluating whether high-dose IV vitamin C infusion (12 g infusion over 4 hours every 12 hours for 7 days) ameliorates the symptoms and progression of severe COVID-19 pneumonia 143.

### Zinc

As previously described, Zn supplementation is considered important for its immune-boosting activities against various viral infections. However, oral supplementation alone is not sufficient to promote Zn availability in infected pneumocytes. Chloroquine and active ingredients of *N. sativa* should be used in combination with Zn to enhance Zn entry into and its effects within pneumocytes. However, it is not clear how to use such a combination or the doses to use in different COVID-19 populations. Black seed oil at doses of 40-80 mg/kg/day is used safely as an adjunctive therapy; however, the Zn recommended daily allowance (RDA) varies according to age, sex, and general health conditions. Zn doses above the RDA are also dangerous because of toxicity. Therefore, future clinical studies on the effects and doses of Zn combined with chloroquine or *N. sativa* in different COVID-19 populations is needed.

## Conclusions

The demographic distribution and severity of the COVID-19 outbreak vary around the world. Whether such distribution is associated with dietary habits, the use of traditional and herbal medicines, differences in geographical distribution or vitamin or mineral deficiencies is still unknown. In this brief review, we discussed traditional health remedies, drugs, vitamins, and minerals with possible use as adjunctive therapies in the COVID-19 era. This is important since we are still experiencing uncontrollable COVID-19 spread and currently have no vaccine or curative medicine. Current evidence does not support the use of such adjunctive treatments in COVID-19; however, we eagerly await results of the rigorous clinical trials on the efficacy and safety of therapies that slow viral spread and improve outcomes.

### What does this article add?

- The treatment of COVID-19 is still unclear until now. However, people can boost their immunity to prevent them from infection after COVID-19 exposure and can reduce their inflammatory reactions to protect their organ deterioration in case suffering from the disease. This review discusses the herbs, medications and nutritional compounds possibly improve their immune systems and regulate the inflammatory reactions related with COVID-19 infection. Although many topics saturated with possible COVID-19 treatments, in this review, we want to convince our readers the importance of COVID-19 interactions with immune cells and inflammatory cells; and further emphasize on the possible pathways related with traditional herbs and nutritional products. Definitely, we need randomized clinical studies to prove the effectiveness of mono or combined therapies. To conclude, we believe that such pathophysiological pathway approach treatment is rational and important for future development of new therapeutic agents for prevention or cure of COVID-19 infection.

## Figures and Tables

**Table 1:** Potential Adjunctive Therapies for COVID-19

Role	Compound
Antiviral activities	Resveratrol, curcumin, vitamin D (ACE2)
	Statins (cholesterol-lowering activity in virus cell membrane)
Anti-inflammatory activities	Vitamin D
	Resveratrol and stilbene-based natural compounds
	Indomethacin
	Iron chelators
Antioxidant activities	Resveratrol and stilbene-based natural compounds
	Vitamin C and zinc
Antithrombotic activities	Statins
Immunomodulatory activities	Vitamin D
	Vitamin C
	Statins
	Melatonin
	Zinc

## References

[1] Di Gennaro F, Pizzol D, Marotta C, Antunes M, Racalbuto V, Veronese N (2020). Coronavirus Diseases (COVID-19) Current Status and Future Perspectives: A Narrative Review. Int J Environ Res Public Health.

[2] Lai CC, Shih TP, Ko WC, Tang HJ, Hsueh PR (2020). Severe acute respiratory syndrome coronavirus 2 (SARS-CoV-2) and coronavirus disease-2019 (COVID-19): The epidemic and the challenges. Int J Antimicrob Agents.

[3] Liu Y, Gayle AA, Wilder-Smith A, Rocklov J (2020). The reproductive number of COVID-19 is higher compared to SARS coronavirus. J Travel Med.

[4] Qian X, Ren R, Wang Y, Guo Y, Fang J, Wu ZD (2020). Fighting against the common enemy of COVID-19: a practice of building a community with a shared future for mankind. Infect Dis Poverty.

[5] van Doremalen N, Bushmaker T, Morris DH, Holbrook MG, Gamble A, Williamson BN (2020). Aerosol and Surface Stability of SARS-CoV-2 as Compared with SARS-CoV-1. N Engl J Med.

[6] Phua J, Weng L, Ling L, Egi M, Lim CM, Divatia JV (2020). Intensive care management of coronavirus disease 2019 (COVID-19): challenges and recommendations. Lancet Respir Med.

[7] Zhou F, Yu T, Du R, Fan G, Liu Y, Liu Z (2020). Clinical course and risk factors for mortality of adult inpatients with COVID-19 in Wuhan, China: a retrospective cohort study. Lancet.

[8] Millet JK, Whittaker GR (2015). Host cell proteases: Critical determinants of coronavirus tropism and pathogenesis. Virus Res.

[9] Gioia M, Ciaccio C, Calligari P, De Simone G, Sbardella D, Tundo G (2020). Role of proteolytic enzymes in the COVID-19 infection and promising therapeutic approaches. Biochem Pharmacol.

[10] Bestle D, Heindl MR, Limburg H, Van Lam van T, Pilgram O, Moulton H (2020). TMPRSS2 and furin are both essential for proteolytic activation of SARS-CoV-2 in human airway cells. Life Sci Alliance.

[11] Banu N, Panikar SS, Leal LR, Leal AR (2020). Protective role of ACE2 and its downregulation in SARS-CoV-2 infection leading to Macrophage Activation Syndrome: Therapeutic implications. Life Sci.

[12] Jafarzadeh A, Chauhan P, Saha B, Jafarzadeh S, Nemati M (2020). Contribution of monocytes and macrophages to the local tissue inflammation and cytokine storm in COVID-19: Lessons from SARS and MERS, and potential therapeutic interventions. Life Sci.

[13] Perico L, Benigni A, Remuzzi G (2020). Should COVID-19 Concern Nephrologists? Why and to What Extent? The Emerging Impasse of Angiotensin Blockade. Nephron.

[14] Qiu Y, Zhao YB, Wang Q, Li JY, Zhou ZJ, Liao CH (2020). Predicting the angiotensin converting enzyme 2 (ACE2) utilizing capability as the receptor of SARS-CoV-2. Microbes Infect.

[15] Bourgonje AR, Abdulle AE, Timens W, Hillebrands JL, Navis GJ, Gordijn SJ (2020). Angiotensin-converting enzyme 2 (ACE2), SARS-CoV-2 and the pathophysiology of coronavirus disease 2019 (COVID-19). J Pathol.

[16] Zou Z, Yan Y, Shu Y, Gao R, Sun Y, Li X (2014). Angiotensin-converting enzyme 2 protects from lethal avian influenza A H5N1 infections. Nat Commun.

[17] Kuba K, Imai Y, Penninger JM (2006). Angiotensin-converting enzyme 2 in lung diseases. Curr Opin Pharmacol.

[18] Imai Y, Kuba K, Rao S, Huan Y, Guo F, Guan B (2005). Angiotensin-converting enzyme 2 protects from severe acute lung failure. Nature.

[19] Wang M, Hao H, Leeper NJ, Zhu L, Early Career C (2018). Thrombotic Regulation From the Endothelial Cell Perspectives. Arterioscler Thromb Vasc Biol.

[20] Zeng H, Pappas C, Belser JA, Houser KV, Zhong W, Wadford DA (2012). Human pulmonary microvascular endothelial cells support productive replication of highly pathogenic avian influenza viruses: possible involvement in the pathogenesis of human H5N1 virus infection. J Virol.

[21] Coperchini F, Chiovato L, Croce L, Magri F, Rotondi M (2020). The cytokine storm in COVID-19: An overview of the involvement of the chemokine/chemokine-receptor system. Cytokine Growth Factor Rev.

[22] Qin C, Zhou L, Hu Z, Zhang S, Yang S, Tao Y (2020). Dysregulation of immune response in patients with COVID-19 in Wuhan, China. Clin Infect Dis.

[23] Huang C, Wang Y, Li X, Ren L, Zhao J, Hu Y (2020). Clinical features of patients infected with 2019 novel coronavirus in Wuhan, China. Lancet.

[24] Yoshikawa T, Hill T, Li K, Peters CJ, Tseng CT (2009). Severe acute respiratory syndrome (SARS) coronavirus-induced lung epithelial cytokines exacerbate SARS pathogenesis by modulating intrinsic functions of monocyte-derived macrophages and dendritic cells. J Virol.

[25] Choe PG, Kang CK, Suh HJ, Jung J, Kang E, Lee SY (2020). Antibody Responses to SARS-CoV-2 at 8 Weeks Postinfection in Asymptomatic Patients. Emerg Infect Dis.

[26] Grifoni A, Weiskopf D, Ramirez SI, Mateus J, Dan JM, Moderbacher CR (2020). Targets of T Cell Responses to SARS-CoV-2 Coronavirus in Humans with COVID-19 Disease and Unexposed Individuals. Cell.

[27] Pia L (2020). SARS-CoV-2-reactive T cells in patients and healthy donors. Nat Rev Immunol.

[28] Xu Z, Shi L, Wang Y, Zhang J, Huang L, Zhang C (2020). Pathological findings of COVID-19 associated with acute respiratory distress syndrome. Lancet Respir Med.

[29] Tian S, Hu W, Niu L, Liu H, Xu H, Xiao SY (2020). Pulmonary Pathology of Early-Phase 2019 Novel Coronavirus (COVID-19) Pneumonia in Two Patients With Lung Cancer. J Thorac Oncol.

[30] Young RE, Thompson RD, Larbi KY, La M, Roberts CE, Shapiro SD (2004). Neutrophil elastase (NE)-deficient mice demonstrate a nonredundant role for NE in neutrophil migration, generation of proinflammatory mediators, and phagocytosis in response to zymosan particles *in vivo*. J Immunol.

[31] Liu S, Su X, Pan P, Zhang L, Hu Y, Tan H (2016). Neutrophil extracellular traps are indirectly triggered by lipopolysaccharide and contribute to acute lung injury. Sci Rep.

[32] Chen G, Wu D, Guo W, Cao Y, Huang D, Wang H (2020). Clinical and immunological features of severe and moderate coronavirus disease 2019. J Clin Invest.

[33] Wang F, Nie J, Wang H, Zhao Q, Xiong Y, Deng L (2020). Characteristics of Peripheral Lymphocyte Subset Alteration in COVID-19 Pneumonia. J Infect Dis.

[34] Fang M, Siciliano NA, Hersperger AR, Roscoe F, Hu A, Ma X (2012). Perforin-dependent CD4+ T-cell cytotoxicity contributes to control a murine poxvirus infection. Proc Natl Acad Sci U S A.

[35] Small BA, Dressel SA, Lawrence CW, Drake DR 3rd, Stoler MH, Enelow RI (2001). CD8(+) T cell-mediated injury *in vivo* progresses in the absence of effector T cells. J Exp Med.

[36] Takeda TA, Sasai M, Adachi Y, Ohnishi K, Fujisawa JI, Izawa S (2017). Potential role of heme metabolism in the inducible expression of heme oxygenase-1. Biochim Biophys Acta Gen Subj.

[37] Fujioka K, Kalish F, Zhao H, Lu S, Wong S, Wong RJ (2017). Induction of Heme Oxygenase-1 Attenuates the Severity of Sepsis in a Non-Surgical Preterm Mouse Model. Shock.

[38] Abouhashem AS, Singh K, Azzazy HME, Sen CK (2020). Is Low Alveolar Type II Cell SOD3 in the Lungs of Elderly Linked to the Observed Severity of COVID-19?. Antioxid Redox Signal.

[39] Knowlton AA, Korzick DH (2014). Estrogen and the female heart. Mol Cell Endocrinol.

[40] Hooper PL, Balogh G, Rivas E, Kavanagh K, Vigh L (2014). The importance of the cellular stress response in the pathogenesis and treatment of type 2 diabetes. Cell Stress Chaperones.

[41] Hooper PL, Hooper JJ (2005). Loss of defense against stress: diabetes and heat shock proteins. Diabetes Technol Ther.

[42] Mehta P, McAuley DF, Brown M, Sanchez E, Tattersall RS, Manson JJ (2020). COVID-19: consider cytokine storm syndromes and immunosuppression. Lancet.

[43] Hsieh YH, Chen CW, Schmitz SF, King CC, Chen WJ, Wu YC (2010). Candidate genes associated with susceptibility for SARS-coronavirus. Bull Math Biol.

[44] Hammad ASA, Ahmed AF, Heeba GH, Taye A (2019). Heme oxygenase-1 contributes to the protective effect of resveratrol against endothelial dysfunction in STZ-induced diabetes in rats. Life Sci.

[45] Riche DM, McEwen CL, Riche KD, Sherman JJ, Wofford MR, Deschamp D (2013). Analysis of safety from a human clinical trial with pterostilbene. J Toxicol.

[46] Kapetanovic IM, Muzzio M, Huang Z, Thompson TN, McCormick DL (2011). Pharmacokinetics, oral bioavailability, and metabolic profile of resveratrol and its dimethylether analog, pterostilbene, in rats. Cancer Chemother Pharmacol.

[47] Aggarwal BB, Gupta SC, Sung B (2013). Curcumin: an orally bioavailable blocker of TNF and other pro-inflammatory biomarkers. Br J Pharmacol.

[48] Moghadamtousi SZ, Kadir HA, Hassandarvish P, Tajik H, Abubakar S, Zandi K (2014). A review on antibacterial, antiviral, and antifungal activity of curcumin. Biomed Res Int.

[49] Fedson DS, Jacobson JR, Rordam OM, Opal SM (2015). Treating the Host Response to Ebola Virus Disease with Generic Statins and Angiotensin Receptor Blockers. mBio.

[50] Lee TS, Chang CC, Zhu Y, Shyy JY (2004). Simvastatin induces heme oxygenase-1: a novel mechanism of vessel protection. Circulation.

[51] Anderson G, Reiter RJ (2020). Melatonin: Roles in influenza, Covid-19, and other viral infections. Rev Med Virol.

[52] Shi S, Lei S, Tang C, Wang K, Xia Z (2019). Melatonin attenuates acute kidney ischemia/reperfusion injury in diabetic rats by activation of the SIRT1/Nrf2/HO-1 signaling pathway. Biosci Rep.

[53] Bravo L (1998). Polyphenols: chemistry, dietary sources, metabolism, and nutritional significance. Nutr Rev.

[54] Gao Y, Fu R, Wang J, Yang X, Wen L, Feng J (2018). Resveratrol mitigates the oxidative stress mediated by hypoxic-ischemic brain injury in neonatal rats via Nrf2/HO-1 pathway. Pharm Biol.

[55] Wu CC, Huang YS, Chen JS, Huang CF, Su SL, Lu KC (2015). Resveratrol ameliorates renal damage, increases expression of heme oxygenase-1, and has anti-complement, anti-oxidative, and anti-apoptotic effects in a murine model of membranous nephropathy. PLoS One.

[56] Wenbin Z, Guojun G (2014). Resveratrol Ameliorates Diabetes-induced Renal Damage through Regulating the Expression of TGF-beta1, Collagen IV and Th17/Treg-related Cytokines in Rats. West Indian Med J.

[57] Diaz-Tocados JM, Rodriguez-Ortiz ME, Almaden Y, Pineda C, Martinez-Moreno JM, Herencia C (2019). Calcimimetics maintain bone turnover in uremic rats despite the concomitant decrease in parathyroid hormone concentration. Kidney Int.

[58] Moussa C, Hebron M, Huang X, Ahn J, Rissman RA, Aisen PS (2017). Resveratrol regulates neuro-inflammation and induces adaptive immunity in Alzheimer's disease. J Neuroinflammation.

[59] Laudati G, Mascolo L, Guida N, Sirabella R, Pizzorusso V, Bruzzaniti S (2019). Resveratrol treatment reduces the vulnerability of SH-SY5Y cells and cortical neurons overexpressing SOD1-G93A to Thimerosal toxicity through SIRT1/DREAM/PDYN pathway. Neurotoxicology.

[60] Ahmed SM, Luo L, Namani A, Wang XJ, Tang X (2017). Nrf2 signaling pathway: Pivotal roles in inflammation. Biochim Biophys Acta Mol Basis Dis.

[61] Klinge CM, Blankenship KA, Risinger KE, Bhatnagar S, Noisin EL, Sumanasekera WK (2005). Resveratrol and estradiol rapidly activate MAPK signaling through estrogen receptors alpha and beta in endothelial cells. J Biol Chem.

[62] Li S, Zhao G, Chen L, Ding Y, Lian J, Hong G (2016). Resveratrol protects mice from paraquat-induced lung injury: The important role of SIRT1 and NRF2 antioxidant pathways. Mol Med Rep.

[63] Jeong J, Juhn K, Lee H, Kim SH, Min BH, Lee KM (2007). SIRT1 promotes DNA repair activity and deacetylation of Ku70. Exp Mol Med.

[64] Yeung ML, Yao Y, Jia L, Chan JF, Chan KH, Cheung KF (2016). MERS coronavirus induces apoptosis in kidney and lung by upregulating Smad7 and FGF2. Nat Microbiol.

[65] Kuroyanagi G, Otsuka T, Yamamoto N, Matsushima-Nishiwaki R, Nakakami A, Mizutani J (2014). Down-regulation by resveratrol of basic fibroblast growth factor-stimulated osteoprotegerin synthesis through suppression of Akt in osteoblasts. Int J Mol Sci.

[66] Pan W, Yu H, Huang S, Zhu P (2016). Resveratrol Protects against TNF-alpha-Induced Injury in Human Umbilical Endothelial Cells through Promoting Sirtuin-1-Induced Repression of NF-KB and p38 MAPK. PLoS One.

[67] Jakus PB, Kalman N, Antus C, Radnai B, Tucsek Z, Gallyas F Jr (2013). TRAF6 is functional in inhibition of TLR4-mediated NF-kappaB activation by resveratrol. J Nutr Biochem.

[68] Wahedi HM, Ahmad S, Abbasi SW (2020). Stilbene-based natural compounds as promising drug candidates against COVID-19. J Biomol Struct Dyn.

[69] Xu J, Zhou L, Weng Q, Xiao L, Li Q (2019). Curcumin analogues attenuate Abeta25-35-induced oxidative stress in PC12 cells via Keap1/Nrf2/HO-1 signaling pathways. Chem Biol Interact.

[70] Prasad S, Tyagi AK (2015). Curcumin and its analogues: a potential natural compound against HIV infection and AIDS. Food Funct.

[71] Praditya D, Kirchhoff L, Bruning J, Rachmawati H, Steinmann J, Steinmann E (2019). Anti-infective Properties of the Golden Spice Curcumin. Front Microbiol.

[72] Mounce BC, Cesaro T, Carrau L, Vallet T, Vignuzzi M (2017). Curcumin inhibits Zika and chikungunya virus infection by inhibiting cell binding. Antiviral Res.

[73] Das S, Sarmah S, Lyndem S, Singha Roy A (2020). An investigation into the identification of potential inhibitors of SARS-CoV-2 main protease using molecular docking study. J Biomol Struct Dyn.

[74] Lelli D, Sahebkar A, Johnston TP, Pedone C (2017). Curcumin use in pulmonary diseases: State of the art and future perspectives. Pharmacol Res.

[75] Wichmann D, Sperhake JP, Lutgehetmann M, Steurer S, Edler C, Heinemann A (2020). Autopsy Findings and Venous Thromboembolism in Patients With COVID-19. Ann Intern Med.

[76] Yimer EM, Tuem KB, Karim A, Ur-Rehman N, Anwar F (2019). Nigella sativa L. (Black Cumin): A Promising Natural Remedy for Wide Range of Illnesses. Evid Based Complement Alternat Med.

[77] Rafati M, Ghasemi A, Saeedi M, Habibi E, Salehifar E, Mosazadeh M (2019). Nigella sativa L. for prevention of acute radiation dermatitis in breast cancer: A randomized, double-blind, placebo-controlled, clinical trial. Complement Ther Med.

[78] Hassanien MF, Assiri AM, Alzohairy AM, Oraby HF (2015). Health-promoting value and food applications of black cumin essential oil: an overview. J Food Sci Technol.

[79] Burits M, Bucar F (2000). Antioxidant activity of Nigella sativa essential oil. Phytother Res.

[80] Salem ML (2005). Immunomodulatory and therapeutic properties of the Nigella sativa L. seed. Int Immunopharmacol.

[81] Majdalawieh AF, Fayyad MW (2015). Immunomodulatory and anti-inflammatory action of Nigella sativa and thymoquinone: A comprehensive review. Int Immunopharmacol.

[82] Singh S, Das SS, Singh G, Schuff C, de Lampasona MP, Catalan CA (2014). Composition, *in vitro* antioxidant and antimicrobial activities of essential oil and oleoresins obtained from black cumin seeds (Nigella sativa L.). Biomed Res Int.

[83] Aljabre SH, Randhawa MA, Akhtar N, Alakloby OM, Alqurashi AM, Aldossary A (2005). Antidermatophyte activity of ether extract of Nigella sativa and its active principle, thymoquinone. J Ethnopharmacol.

[84] Salem ML, Hossain MS (2000). Protective effect of black seed oil from Nigella sativa against murine cytomegalovirus infection. Int J Immunopharmacol.

[85] Boskabady MH, Keyhanmanesh R, Khameneh S, Doostdar Y, Khakzad MR (2011). Potential immunomodulation effect of the extract of Nigella sativa on ovalbumin sensitized guinea pigs. J Zhejiang Univ Sci B.

[86] Barakat EM, El Wakeel LM, Hagag RS (2013). Effects of Nigella sativa on outcome of hepatitis C in Egypt. World J Gastroenterol.

[87] Ahmad S, Abbasi HW, Shahid S, Gul S, Abbasi SW (2020). Molecular docking, simulation and MM-PBSA studies of nigella sativa compounds: a computational quest to identify potential natural antiviral for COVID-19 treatment. J Biomol Struct Dyn.

[88] Denison MR, Zoltick PW, Hughes SA, Giangreco B, Olson AL, Perlman S (1992). Intracellular processing of the N-terminal ORF 1a proteins of the coronavirus MHV-A59 requires multiple proteolytic events. Virology.

[89] te Velthuis AJ, van den Worm SH, Sims AC, Baric RS, Snijder EJ, van Hemert MJ (2010). Zn(2+) inhibits coronavirus and arterivirus RNA polymerase activity *in vitro* and zinc ionophores block the replication of these viruses in cell culture. PLoS Pathog.

[90] Chansrichavala P, Chantharaksri U, Sritara P, Chaiyaroj SC (2009). Atorvastatin attenuates TLR4-mediated NF-kappaB activation in a MyD88-dependent pathway. Asian Pac J Allergy Immunol.

[91] Albert MA, Danielson E, Rifai N, Ridker PM, Investigators P (2001). Effect of statin therapy on C-reactive protein levels: the pravastatin inflammation/CRP evaluation (PRINCE): a randomized trial and cohort study. JAMA.

[92] Vandermeer ML, Thomas AR, Kamimoto L, Reingold A, Gershman K, Meek J (2012). Association between use of statins and mortality among patients hospitalized with laboratory-confirmed influenza virus infections: a multistate study. J Infect Dis.

[93] Fedson DS (2013). Treating influenza with statins and other immunomodulatory agents. Antiviral Res.

[94] Laidler MR, Thomas A, Baumbach J, Kirley PD, Meek J, Aragon D (2015). Statin treatment and mortality: propensity score-matched analyses of 2007-2008 and 2009-2010 laboratory-confirmed influenza hospitalizations. Open Forum Infect Dis.

[95] Rogers AJ, Guan J, Trtchounian A, Hunninghake GM, Kaimal R, Desai M (2019). Association of Elevated Plasma Interleukin-18 Level With Increased Mortality in a Clinical Trial of Statin Treatment for Acute Respiratory Distress Syndrome. Crit Care Med.

[96] Rosen RJ (2020). Thrombotic complications in critically ill patients with COVID 19. Thromb Res.

[97] Klok FA, Kruip M, van der Meer NJM, Arbous MS, Gommers D, Kant KM (2020). Incidence of thrombotic complications in critically ill ICU patients with COVID-19. Thromb Res.

[98] Helms J, Tacquard C, Severac F, Leonard-Lorant I, Ohana M, Delabranche X (2020). High risk of thrombosis in patients with severe SARS-CoV-2 infection: a multicenter prospective cohort study. Intensive Care Med.

[99] Glynn RJ, Danielson E, Fonseca FA, Genest J, Gotto AM Jr, Kastelein JJ (2009). A randomized trial of rosuvastatin in the prevention of venous thromboembolism. N Engl J Med.

[100] Arslan F, Pasterkamp G, de Kleijn DP (2008). Unraveling pleiotropic effects of statins: bit by bit, a slow case with perspective. Circ Res.

[101] Srinivasan V, Maestroni GJ, Cardinali DP, Esquifino AI, Perumal SR, Miller SC (2005). Melatonin, immune function and aging. Immun Ageing.

[102] Sundberg I, Rasmusson AJ, Ramklint M, Just D, Ekselius L, Cunningham JL (2020). Daytime melatonin levels in saliva are associated with inflammatory markers and anxiety disorders. Psychoneuroendocrinology.

[103] Lin GJ, Huang SH, Chen SJ, Wang CH, Chang DM, Sytwu HK (2013). Modulation by melatonin of the pathogenesis of inflammatory autoimmune diseases. Int J Mol Sci.

[104] Wu GC, Peng CK, Liao WI, Pao HP, Huang KL, Chu SJ (2020). Melatonin receptor agonist protects against acute lung injury induced by ventilator through up-regulation of IL-10 production. Respir Res.

[105] Petrovsky N, Harrison LC (1998). The chronobiology of human cytokine production. Int Rev Immunol.

[106] Carrillo-Vico A, Reiter RJ, Lardone PJ, Herrera JL, Fernandez-Montesinos R, Guerrero JM (2006). The modulatory role of melatonin on immune responsiveness. Curr Opin Investig Drugs.

[107] Hardeland R (2019). Aging, Melatonin, and the Pro- and Anti-Inflammatory Networks. Int J Mol Sci.

[108] Gunn PJ, Middleton B, Davies SK, Revell VL, Skene DJ (2016). Sex differences in the circadian profiles of melatonin and cortisol in plasma and urine matrices under constant routine conditions. Chronobiol Int.

[109] Amaral FGD, Cipolla-Neto J (2018). A brief review about melatonin, a pineal hormone. Arch Endocrinol Metab.

[110] Chmiel JF, Konstan MW, Accurso FJ, Lymp J, Mayer-Hamblett N, VanDevanter DR (2015). Use of ibuprofen to assess inflammatory biomarkers in induced sputum: Implications for clinical trials in cystic fibrosis. J Cyst Fibros.

[111] Rossen JW, Bouma J, Raatgeep RH, Buller HA, Einerhand AW (2004). Inhibition of cyclooxygenase activity reduces rotavirus infection at a postbinding step. J Virol.

[112] Reynolds AE, Enquist LW (2006). Biological interactions between herpesviruses and cyclooxygenase enzymes. Rev Med Virol.

[113] Schroer J, Shenk T (2008). Inhibition of cyclooxygenase activity blocks cell-to-cell spread of human cytomegalovirus. Proc Natl Acad Sci U S A.

[114] Bahrami H, Daryani NE, Haghpanah B, Moayyeri A, Moghadam KF, Mirmomen S (2005). Effects of indomethacin on viral replication markers in asymptomatic carriers of hepatitis B: a randomized, placebo-controlled trial. Am J Gastroenterol.

[115] Amici C, Di Caro A, Ciucci A, Chiappa L, Castilletti C, Martella V (2006). Indomethacin has a potent antiviral activity against SARS coronavirus. Antivir Ther.

[116] Gupta R, Misra A (2020). Contentious issues and evolving concepts in the clinical presentation and management of patients with COVID-19 infectionwith reference to use of therapeutic and other drugs used in Co-morbid diseases (Hypertension, diabetes etc). Diabetes Metab Syndr.

[117] Day M (2020). Covid-19: ibuprofen should not be used for managing symptoms, say doctors and scientists. BMJ.

[118] Ali MK, Kim RY, Brown AC, Donovan C, Vanka KS, Mayall JR (2020). Critical role for iron accumulation in the pathogenesis of fibrotic lung disease. J Pathol.

[119] Turi JL, Yang F, Garrick MD, Piantadosi CA, Ghio AJ (2004). The iron cycle and oxidative stress in the lung. Free Radic Biol Med.

[120] Chen N, Zhou M, Dong X, Qu J, Gong F, Han Y (2020). Epidemiological and clinical characteristics of 99 cases of 2019 novel coronavirus pneumonia in Wuhan, China: a descriptive study. Lancet.

[121] Kell DB, Pretorius E (2014). Serum ferritin is an important inflammatory disease marker, as it is mainly a leakage product from damaged cells. Metallomics.

[122] Williams A, Meyer D (2009). Desferrioxamine as immunomodulatory agent during microorganism infection. Curr Pharm Des.

[123] Visseren F, Verkerk MS, van der Bruggen T, Marx JJ, van Asbeck BS, Diepersloot RJ (2002). Iron chelation and hydroxyl radical scavenging reduce the inflammatory response of endothelial cells after infection with Chlamydia pneumoniae or influenza A. Eur J Clin Invest.

[124] Schleicher RL, Sternberg MR, Looker AC, Yetley EA, Lacher DA, Sempos CT (2016). National Estimates of Serum Total 25-Hydroxyvitamin D and Metabolite Concentrations Measured by Liquid Chromatography-Tandem Mass Spectrometry in the US Population during 2007-2010. J Nutr.

[125] Kara M, Ekiz T, Ricci V, Kara O, Chang KV, Ozcakar L (2020). 'Scientific Strabismus' or two related pandemics: coronavirus disease and vitamin D deficiency. Br J Nutr.

[126] Khachfe HH, Chahrour M, Sammouri J, Salhab H, Makki BE, Fares M (2020). An Epidemiological Study on COVID-19: A Rapidly Spreading Disease. Cureus.

[127] Shultz JM, Perlin A, Saltzman RG, Espinel Z, Galea S (2020). Pandemic March: 2019 Coronavirus Disease's First Wave Circumnavigates the Globe. Disaster Med Public Health Prep.

[128] Holmes L Jr, Enwere M, Williams J, Ogundele B, Chavan P, Piccoli T (2020). Black-White Risk Differentials in COVID-19 (SARS-COV2) Transmission, Mortality and Case Fatality in the United States: Translational Epidemiologic Perspective and Challenges. Int J Environ Res Public Health.

[129] Ferdinand KC, Nasser SA (2020). African-American COVID-19 Mortality: A Sentinel Event. J Am Coll Cardiol.

[130] Harris SS (2006). Vitamin D and African Americans. J Nutr.

[131] Looker AC, Dawson-Hughes B, Calvo MS, Gunter EW, Sahyoun NR (2002). Serum 25-hydroxyvitamin D status of adolescents and adults in two seasonal subpopulations from NHANES III. Bone.

[132] Zheng JQ, Hou YC, Zheng CM, Lu CL, Liu WC, Wu CC (2016). Cholecalciferol Additively Reduces Serum Parathyroid Hormone and Increases Vitamin D and Cathelicidin Levels in Paricalcitol-Treated Secondary Hyperparathyroid Hemodialysis Patients. Nutrients.

[133] Liu WC, Zheng CM, Lu CL, Lin YF, Shyu JF, Wu CC (2015). Vitamin D and immune function in chronic kidney disease. Clin Chim Acta.

[134] Ahmed A, Siman-Tov G, Keck F, Kortchak S, Bakovic A, Risner K (2019). Human cathelicidin peptide LL-37 as a therapeutic antiviral targeting Venezuelan equine encephalitis virus infections. Antiviral Res.

[135] Pachon-Ibanez ME, Smani Y, Pachon J, Sanchez-Cespedes J (2017). Perspectives for clinical use of engineered human host defense antimicrobial peptides. FEMS Microbiol Rev.

[136] Findlay F, Proudfoot L, Stevens C, Barlow PG (2016). Cationic host defense peptides; novel antimicrobial therapeutics against Category A pathogens and emerging infections. Pathog Glob Health.

[137] Xu J, Yang J, Chen J, Luo Q, Zhang Q, Zhang H (2017). Vitamin D alleviates lipopolysaccharideinduced acute lung injury via regulation of the reninangiotensin system. Mol Med Rep.

[138] Meltzer DO, Best TJ, Zhang H, Vokes T, Arora V, Solway J (2020). Association of Vitamin D Status and Other Clinical Characteristics With COVID-19 Test Results. JAMA Netw Open.

[139] Mitchell F (2020). Vitamin-D and COVID-19: do deficient risk a poorer outcome?. Lancet Diabetes Endocrinol.

[140] Grant WB, Lahore H, McDonnell SL, Baggerly CA, French CB, Aliano JL (2020). Evidence that Vitamin D Supplementation Could Reduce Risk of Influenza and COVID-19 Infections and Deaths. Nutrients.

[141] Colunga Biancatelli RML, Berrill M, Marik PE (2020). The antiviral properties of vitamin C. Expert Rev Anti Infect Ther.

[142] Douglas RM, Chalker EB, Treacy B (2000). Vitamin C for preventing and treating the common cold. Cochrane Database Syst Rev.

[143] Wu R, Wang L, Kuo HD, Shannar A, Peter R, Chou PJ (2020). An Update on Current Therapeutic Drugs Treating COVID-19. Curr Pharmacol Rep.

